# Lack of online price transparency of small animal veterinary clinics in the United States

**DOI:** 10.3389/fvets.2026.1788878

**Published:** 2026-05-04

**Authors:** Alexis Adams, Alexandra Gilley, Victoria Morris, Lauren Wisnieski

**Affiliations:** Richard A. Gillespie College of Veterinary Medicine, Lincoln Memorial University, Harrogate, TN, United States

**Keywords:** access to care, companion animal, price transparency, small animal, veterinary medicine

## Abstract

**Introduction:**

The cost of veterinary care is a significant concern for many pet owners. Despite the growing use of the internet for service-related information, there is limited research regarding the accessibility of veterinary pricing information on veterinary clinic websites.

**Materials and methods:**

To address this gap, the present study investigated the transparency of online veterinary service pricing on websites for small animal clinics within the United States through a cross-sectional study design. The country was divided into four divisions based on the U.S. Census, with two randomly selected states selected from each division (Wyoming, California, Michigan, Illinois, Maine, New York, Florida, and Kentucky). Systematic random sampling within state was used to select five distinct zip codes, comprising of two urban, two rural, and one suburban area per Census definitions. The first five clinics within a specific zip code on Google Maps search results were included for analysis. Prices for routine dog and cat services were extracted from clinic websites. These included core vaccinations, sterilization procedures (spay and neuter surgeries), nail trims, dental procedures, and “Wellness Plans.”

**Results:**

Although a maximum of five clinics per zip code were targeted, resulting in an estimated sample size of 200 clinics, some of the selected zip codes had fewer than five clinics, resulting in a final size of 177 clinics. Among these, 157 clinics (88.70%, 157/177) had websites available for review, demonstrating a high level of online presence. However, information on specific service pricing was sparse. Three clinics (1.91%, 3/157) provided pricing information for services on their websites. Similarly, only seven clinics (4.46%, 7/157) offered a Wellness Plan option for patients, 2/157 (1.27%) clinics mentioned insurance options, 13/157 (8.28%) clinics listed payment options such as CareCredit, Scratch pay, check, cash, and 14/157 (8.92%) clinics offered discounts such as free or reduced cost first visit fees.

**Discussion:**

These findings highlight a substantial gap in online price transparency, underscoring the need for increased transparency to promote informed decision-making and trust between clients and veterinarians.

## 1 Introduction

In human healthcare, efforts to improve price transparency have aimed to empower patients, foster competition, and enhance the overall efficiency of healthcare systems (1). Indeed, there have been multiple research studies investigating price transparency in human medicine (1–4). For example, Miller et al. (3) found that healthcare transparency is increasingly viewed as a key policy mechanism in the United States to enhance market competition, lower healthcare costs, and improve access to affordable, high-quality care (3). These issues are met with the identified need for stricter compliance measures, streamlined regulations, and clear, patient-centered quality metrics to enhance price shopping, and informed decision-making (3). However, despite the ubiquitous research on price transparency in human medicine, comparable research in veterinary medicine remains sparse.

Internationally, veterinary pricing transparency varies substantially by regulatory framework. In the United States, there are no federal requirements mandating public disclosure of veterinary fees, and professional guidance from the American Veterinary Medical Association (AVMA) emphasizes clear communication of costs and provision of estimates rather than published price lists.^1^^,^^2^ In contrast, some countries have more structured or legally mandated pricing systems; for example, Germany regulates veterinary fees through the *Gebührenordnung für Tierärzte (GOT)*, which provides standardized fee schedules.^3^ More recently, the United Kingdom has introduced regulatory reforms following a Competition and Markets Authority (CMA) investigation, including requirements for published price lists and enhanced price transparency for veterinary services.^4^

Veterinary practices may be hesitant to publish prices online due to the inherent variability in clinical care and the difficulty of standardizing costs across patients and procedures, as well as the need for frequent price adjustments (5). Additionally, treatment plans may evolve based on patient response or complications, meaning initial estimates may not reflect final costs, which can contribute to client dissatisfaction when expenses exceed expectations.

Veterinary service prices in the United States have increased substantially over time, with periods in recent years in which veterinary-specific inflation has exceeded broader economy-wide inflation, leading to growing concerns about affordability among pet owners (6, 7). Recent analyses have highlighted that rising expenditures on companion animal health care, driven in part by advances in diagnostics and treatment intensity, have become a significant financial burden for many households and have been widely discussed in both academic and policy contexts. Pricing transparency can help mitigate the financial burden of unpaid invoices, which can impact a clinic’s sustainability and patient access to care (5). This, in turn, gives the client a full understanding of options so they can make an informed healthcare decision about their pet’s spectrum of care (8). In addition, veterinarians can better communicate the value of their services and discuss treatment options to align with the pet owners’ financial capabilities (9).

To the best of our knowledge, there is only one study that estimated online price transparency in veterinary clinics. This study was conducted in Europe (Sweden, Denmark, Norway, and the United Kingdom) and examined the prices of veterinary care for dogs, cats, and horses (5). This research focused on collecting price data directly from veterinary clinics and comparing it with a veterinary price comparison website. The study found that the price information was often incomplete or inconsistently presented on clinical websites, which makes it harder for clients to make informed decisions (5). Moreover, many clinics failed to provide basic pricing for common procedures, and the price comparison tool only partially aligned with the clinic’s actual prices (5, 10). This limits standardization of prices and the public’s accessibility to cost information. The objective of this study was to evaluate the prevalence of transparency of online veterinary service pricing across small animal clinics within the United States. Specifically, it aimed to determine how frequently clinics provide cost information for routine services on their websites, and identify any patterns in payment options, “Wellness Plans,” or discount offerings.

## 2 Materials and methods

A list of veterinary services of interest was developed *a priori* to guide data extraction. Following a preliminary review of a subset of veterinary practice websites, additional variables related to financial accessibility, specifically payment options and the availability of payment plans, were incorporated into the data extraction framework. The final list included prices regarding various services (physical exams, core vaccinations, sterilization procedures, dental services, nail trims, and Wellness Plans) and any information on payment options, insurance options, or payment plans. Data were collected directly from publicly available information on veterinary practice websites. While service categories were initially expected to emerge from the data, no predefined classification scheme was established prior to data collection; instead, categories were developed iteratively based on the services identified during website review. We initially aimed to extract cost information stratified by species (e.g., dog versus cat). However, due to limited and inconsistent reporting of species-specific pricing across websites, this level of detail could not be reliably obtained and was therefore not included in the final analysis.

An estimated sample size of 200 veterinary clinics was calculated in OpenEpi based on a predicted prevalence of 15%, with 95% confidence, and 5% absolute precision (11). A website with a randomization tool was used to select two states from each Census region of the U.S.: Northeast, Midwest, South, and West.^5^ For each selected state, systematic random sampling was used to select two urban zip codes, two rural zip codes, and one suburban zip code based on U.S. Census definitions.^6^ Small animal veterinary clinics were identified by searching “vet clinics in [zip code]” within Google Maps.^7^ Within the search results, the top five small animal veterinary clinics that were listed within a specific zip code were included in the study.

Due to the low number of veterinary clinic websites with pricing information (*n* = 3), only descriptive information was summarized for service prices.

## 3 Results

In this study, we examined veterinary clinic service pricing within eight states: Wyoming, California, Michigan, Illinois, Maine, New York, Florida, and Kentucky. Due to some zip codes having less than five clinics, the final sample size was 177 clinics (Table 1). Among the identified clinics, 157/177 (88.7%) had websites to be reviewed. However, information regarding specific services was limited. Only 3/157 (1.91%), listed pricing information on their websites (Table 2). The few clinics that included prices typically listed costs for services such as wellness exams or dental cleanings. More complex procedures or diagnostic services prices were not listed online.

**Table 1:** Availability of veterinary clinic websites and online pricing information by United States region and state (*N* = 177).

Region	State	Total clinics included	Clinics with websites (*n*[%])	Clinics with online pricing that have websites (*n*[%])
Midwest	IL	19	14 (73.7)	0 (0.00)
	MI	21	17 (80.9)	0 (0.00)
Northeast	ME	25	24 (96.0)	1 (4.16)
	NY	22	21 (95.5)	1 (4.76)
South	FL	25	24 (96.0)	1 (4.16)
	KY	22	18 (81.8)	0 (0.00)
West	CA	23	22 (95.6)	0 (0.00)
	WY	20	17 (85.0)	0 (0.00)

**Table 2:** Resulting veterinary service prices by region, state, and clinic location type within the United States (*N* = 3).

Region	State	Urban/Rural/Suburban	Rabies	Physical exam	Core vaccines	Dentals	Nail Trim	Spay/Neuter
Northeast	ME	Urban	–	$135	–	–	–	–
	NY	Suburban	–	$45	–	–	–	–
South	FL	Urban	–	$29	–	$349	–	–

In contrast, some clinics offered alternative options to assist clients in managing the cost of their pet’s veterinary care. For example, 7/157 (4.46%) of clinics provided Wellness Plan package options to clients, which bundled preventive care services into monthly payments. The composition of Wellness Plans varied among clinics: some offered core vaccines and annual bloodwork, others provided only core vaccinations, and one clinic included core vaccinations, annual bloodwork, and nail trims. Additionally, 2/157 (1.27%) clinics mentioned insurance options on their website. In terms of payment methods, 13/157 (8.28%) clinics described some method of payment on their website. Many offered third-party payment options such as CareCredit and Scratch Pay, allowing pet owners to pay for treatments over time. Standard payment methods, such as credit cards, PayPal, cash, and checks were commonly mentioned; however, these do not provide financing and require full payments at the time of service. Additionally, 14/157 (8.92%) clinics offered discounts, including percentage discounts (10–30%), free first visits, or reduced first visit fees.

## 4 Discussion

There is limited research on price transparency in veterinary medicine, despite the significant progress in human medicine. An online presence is important for businesses, including veterinary clinics (12, 13). Many pet owners rely on the internet to find information about local veterinarians, services offered, and associated costs (13). However, despite the prevalence of veterinary clinic websites, the availability of detailed service information is relatively unknown among veterinary clinics. To the best of our knowledge, this is the first investigation of the availability of U.S. small animal veterinary pricing information online, offering important insights into a topic that has remained largely unexplored. These findings are best interpreted within the context of differing regulatory frameworks governing veterinary pricing transparency across countries. In the United States, veterinary pricing and advertising practices are largely self-regulated, with no federal requirements mandating public disclosure of fees. Professional guidance emphasizes transparent communication of costs between veterinarians and clients but does not require standardized publication of price lists or regulate promotional pricing strategies. In contrast, several international frameworks adopt more structured approaches, such as Germany’s *GOT* (see footnote 3), which establishes standardized fee schedules for veterinary services. More recently, the United Kingdom’s CMA (see footnote 4) has introduced reforms aimed at increasing price transparency, including requirements for clearer and more accessible publication of veterinary fees. These differing regulatory environments highlight how policy structures may shape the availability of pricing information and consumer access to cost data in veterinary care. Egenvall et al. (5) analyzed data on prices from veterinary clinic websites in Europe and noted that while some online prices existed, this information is often unclear, inconsistent, or missing altogether, particularly in European countries where veterinary clinics are not required to publicly publish their service prices. The authors emphasized the need for improved transparency.

Overall, our results highlighted that while a significant portion of veterinary clinics have a website, detailed service information such as pricing and Wellness Plans are not commonly available. When pet owners cannot find clear information about services and costs online, it hinders their ability to compare veterinary clinic pricing. Additionally, studies such as Kipperman et al. (14) have highlighted that veterinarians often feel discomfort discussing costs due to concerns about client reactions, fear of being perceived as profit-driven, or uncertainty about a client’s financial situation. Many owners feel uncertain about what to expect financially, which could delay future necessary veterinary visits or lead to unexpected expenses (10). Transparency in pricing and service details fosters trust between clinics and pet owners (5). By providing accessible information online, veterinary clinics can establish a stronger relationship with clients demonstrating their commitment to transparency and care. As a result, clinics can increase their client base, foster loyalty, and improve overall revenue by encouraging more regular visits for preventative care and wellness services (15, 16). Additionally, pricing transparency plays a critical role in supporting the spectrum of care, a concept that emphasizes offering a range of diagnostic treatment options to accommodate different financial needs of a client. When clinics clearly communicate service costs, clients can make informed decisions about their pet’s care, helping bridge the gap between affordability and quality treatment (17). Online price listings, cost estimators, or Wellness Plans can guide pet owners towards appropriate care options within their budget, reducing the likelihood of financial surprises (18). Clinics that offer payment plans, Wellness Plans, or information on pet insurance and alternative payment options (e.g., CareCredit) can differentiate themselves from other clinics in an increasingly competitive market. Wellness Plans are a means of providing essential preventative care, yet few clinics detail these options online (18). Clearly listing service price ranges, offering Wellness Plans, and providing information on payment options, insurance, and discounts could help attract more clients and encourage participation in these programs (18, 19). Likewise, transparency about the methods of payment accepted or third-party financing tools such as CareCredit or pet insurance may provide the opportunity to alleviate cost-related hesitation among pet-owners. This can make veterinary services more accessible and improve the continuity of care by encouraging proactive financial planning and increasing perceived affordability of routine services (9, 18).

One of the key strengths of our study is that it provides the first comprehensive look at small animal veterinary clinic online price transparency across the entire U.S. Our data includes rural, urban, and suburban veterinary practices, offering a broad perspective on how different types of clinics handle price communication (20). This may not represent global trends but can provide an estimate for national veterinary pricing transparency trends. However, our study had several limitations. First, we were unable to calculate differences in pricing by veterinary clinic location due to the low number of clinics with prices available. Also, an additional limitation is that we used the top five veterinary clinic results from Google Maps, which may not be truly representative of the zip codes they were sampled from. Data extraction was conducted by a single reviewer and was not independently validated, which may increase the risk of measurement error. We also did not assess the last updated date of clinic websites or pricing pages; therefore, some information may not reflect current pricing or service offerings, and future studies should consider incorporating website update timestamps to improve data currency and accuracy. Our results reflect services and pricing advertised online and may underestimate what is offered in practice (e.g., wellness plans). Future studies incorporating in-practice or phone-based data collection could better characterize gaps between online information and actual service availability. In-practice or phone-based data collection should also be incorporated in future studies to collect clinic-level operational characteristics such as ownership structure (e.g., corporate vs. independent), years in operation, or staffing levels. These factors may influence pricing transparency and digital presence but were beyond the scope of the current study, which focused on publicly available online information.

In conclusion, while many veterinary clinics maintain an online presence, providing service prices is an unexplored opportunity There is significant potential for future research on price transparency in veterinary medicine. Future studies could expand on this work by exploring how different pricing models (i.e., flat rate vs. flexible pricing) could impact both the client’s behavior and clinic profitability. Additionally, future studies could explore how online price lists or cost calculators enhance the transparency of these prices without compromising the flexibility that is often required in veterinary care. Research could explore how such systems impact client decision-making, clinic operations, and overall accessibility of veterinary care. Additionally, studies could examine how to balance transparency with the need for individualized pricing, ensuring that online tools provide useful guidance without oversimplifying the complexities of veterinary services.

## Data Availability

The raw data supporting the conclusions of this article will be made available by the authors, without undue reservation.

## Footnotes

1
https://www.avma.org/resources-tools/practice-management/pricing-strategy-veterinary-practices

2
https://www.avma.org/resources-tools/practice-management/communicating-clients-using-right-language-improve-care

3
https://www.bmleh.de/DE/themen/tiere/got.html

4 https://www.gov.uk/government/news/cma-concludes-market-investigation-with-major-reforms-to-veterinary-sector?

5
http://www.randomizer.org

6
https://www.census.gov/programs-surveys/geography/guidance/geo-areas/urban-rural.html

7
https://www.google.com/maps

## References

[1] Pollack HA. Necessity for and limitations of price transparency in American health care. AMA J Ethics. (2022) 24:e1069–74. doi: 10.1001/amajethics.2022.1069 36342490

[2] Jiang JX Krishnan R Bai G. Price transparency in hospitals—current research and future directions. JAMA Netw Open. (2023) 6:e2249588. doi: 10.1001/jamanetworkopen.2022.49588 36602805

[3] Miller BJ Mandelberg MC Griffith NC Ehrenfeld JM. Price transparency: empowering patient choice and promoting provider competition. J Med Syst. (2020) 44:80. doi: 10.1007/s10916-020-01553-2, 32140942

[4] Rao P Fischer SH Vaiana ME Taylor EA. Barriers to price and quality transparency in health care markets. RAND Health Q. (2022) 9:1. 35837511 10.7249/rhq-09-03-01 PMC9242565

[5] Egenvall A Höglund OV Hoffman R Valle PS Andersen PH Lönnell C . Prices for veterinary care of dogs, cats and horses in selected countries in Europe. Front Vet Sci. (2024) 11:1403483. doi: 10.3389/fvets.2024.1403483, 39091400 PMC11292583

[6] Bir C Pasteur K Widmar NO Croney CC. Pet acquisition trends and veterinary care access in the United States. PLoS One. (2025) 20:e0325075. doi: 10.1371/journal.pone.0325075 40601616 PMC12220994

[7] Neill CL Salois M McKay C. Anticipating the downturn: business cycle forecasting for veterinary practice strategy in the United States. Front Vet Sci. (2025) 12:1689704. doi: 10.3389/fvets.2025.1689704, 41180239 PMC12571561

[8] Klingborg DJ Klingborg J. Talking with veterinary clients about money. Vet Clin Small Anim. (2007) 37:79–93. doi: 10.1016/j.cvsm.2006.09.007, 17162113

[9] Robinson R. Pricing. Part 1: understanding pricing structures in veterinary medicine. Pract Manag. (2025) 47:368–70. doi: 10.1002/inpr.570

[10] Groves CNH Janke N Stroyev A Tayce JD Coe JB. Discussion of cost continues to be uncommon in companion animal veterinary practice. J Am Vet Med Assoc. (2022) 260:1844–52. doi: 10.2460/javma.22.06.0268, 36074746

[11] Sullivan KM Dean A Soe MM. OpenEpi: a web-based epidemiologic and statistical calculator for public health. Public Health Rep. (2009) 124:471–4. doi: 10.1177/003335490912400320, 19445426 PMC2663701

[12] DeWilde C. Social media and digital marketing for veterinary practices. Vet Clin Small Anim. (2024) 54:381–94. doi: 10.1016/j.cvsm.2023.10.006 37996301

[13] Kogan L Oxley JA Hellyer P Schoenfeld R Rishniw M. UK pet owners’ use of the internet for online pet health information. Vet Rec. (2018) 182:601. doi: 10.1136/vr.104716, 29549181

[14] Kipperman BS Kass PH Rishniw M. Factors that influence small animal veterinarians’ opinions and actions regarding cost of care and effects of economic limitations on patient care and outcome and professional career satisfaction and burnout. J Am Vet Med Assoc. (2017) 250:785–94. doi: 10.2460/javma.250.7.785, 28306486

[15] Beyer K. Unlocking the potential of ICT innovation in veterinary healthcare: the pathway to improve practices and business model. Procedia Comput Sci. (2023) 225:4775–84. doi: 10.1016/j.procs.2023.10.477

[16] Sutherland KA Coe JB Blais-Vaillancourt K Gober M Berst H. Veterinary clients prefer benefit-focused online communication while clinic websites uncommonly communicate benefits of preventive care services. J Am Vet Med Assoc. (2025) 263:113–21. doi: 10.2460/javma.24.09.0568, 39488076

[17] Brown CR Garrett LD Gilles WK Houlihan KE McCobb E Pailler S . Spectrum of care: more than treatment options. J Am Vet Med Assoc. (2021) 259:712–7. doi: 10.2460/javma.259.7.712 34516261

[18] Volk JO Hartmann G. How wellness plans grow veterinary practice. J Am Vet Med Assoc. (2015) 247:40–1. doi: 10.2460/javma.247.1.40, 26086224

[19] Robinson R. Pricing. Part 2: charging for veterinary expertise and handling grey areas – building trust through transparency. Pract Manag. (2025) 47:434–8. doi: 10.1002/inpr.70004

[20] Villarroel A McDonald S Walker W Kaiser L Dewell R Dewell G. Shortage of rural veterinarians: real or perceived? Online J Rural Res Policy. (2010) 5:1–11. doi: 10.4148/ojrrp.v5i7.269

